# Conscious Wireless Electroretinogram and Visual Evoked Potentials in Rats

**DOI:** 10.1371/journal.pone.0074172

**Published:** 2013-09-12

**Authors:** Jason Charng, Christine T. Nguyen, Zheng He, Trung M. Dang, Algis J. Vingrys, Rebecca L. Fish, Rachel Gurrell, Phil Brain, Bang V. Bui

**Affiliations:** 1 Department of Optometry & Vision Sciences, University of Melbourne, Victoria, Australia; 2 Pfizer Neusentis, Cambridge, United Kingdom; 3 Pfizer Global Research and Development, Kent, United Kingdom; University of Houston, United States of America

## Abstract

The electroretinogram (ERG, retina) and visual evoked potential (VEP, brain) are widely used *in vivo* tools assaying the integrity of the visual pathway. Current recordings in preclinical models are conducted under anesthesia, which alters neural physiology and contaminates responses. We describe a conscious wireless ERG and VEP recording platform in rats. Using a novel surgical technique to chronically implant electrodes subconjunctivally on the eye and epidurally over the visual cortex, we are able to record stable and repeatable conscious ERG and VEP signals over at least 1 month. We show that the use of anaesthetics, necessary for conventional ERG and VEP measurements, alters electrophysiology recordings. Conscious visual electrophysiology improves the viability of longitudinal studies by eliminating complications associated with repeated anaesthesia. It will also enable uncontaminated assessment of drug effects, allowing the eye to be used as an effective biomarker of the central nervous system.

## Introduction

The electroretinogram (ERG) and the visual evoked potential (VEP) are widely used non-invasive tools in laboratory and clinical settings. These techniques allow real-time, *in vivo* functional assays of retina (ERG) and visual cortex (VEP) on light stimulation. A key advantage of the ERG is that the composite waveform can be broken down to components, each reflecting the integrity of particular retinal cell classes [1,2]. Of the various stimuli used to elicit the ERG, a full-field flash provides the most comprehensive index of global retinal function. The major components of the flash ERG comprise an initial negative waveform (a-wave; photoreceptor response) followed by a large positivity (b-wave; bipolar cell response), which can be further broken down to rod (night vision) or cone (day vision) contributions [3]. Early components of the VEP (P1-N1) reflect the integrity of the optic nerve [4], whereas latter components reflect processing by higher cortical centers (P2 onwards) [5,6]. Hence simultaneous measurement of the ERG and VEP allows comprehensive assessment of different serial elements along the visual pathway.

To date, ERG and VEP measurements in animals require general anaesthesia, which is known to modify body temperature [7], blood flow [8] and heart rate [9]. As anaesthetic compounds exert their sedative and analgesic effects by acting on neurochemical receptors in the central nervous system [10,11], it is not surprising that both retinal [12] and cortical [13] responses are also altered. Of the commonly used laboratory anaesthetics, ketamine acts mainly on N-methyl d-aspartate (NMDA) receptors [14], xylazine on α_2_ receptors [15] and isoflurane on NMDA [16], gamma-aminobutyric acid (GABA) [17] and glycine [18] receptors, all of which are localized in both retina and brain. This confound greatly limits the use of the anaesthetized ERG and VEP as biomarkers for preclinical drug testing that target these receptor systems. Other disadvantages of anaesthesia are that it limits the duration of experiments, as prolonged or repeated anaesthesia can lead to perioperative respiratory complications [19]. More importantly, anaesthetic usage does not replicate conscious clinical recordings.

There have been attempts to measure awake ERG and VEPs via tethered setups, however these studies have several shortcomings [11,20-22]. Specifically, the use of wire connectors restrict animal movement and natural behaviour leading to stress confounds [23]. Tang and colleagues [24] have reported that differences in weight and flexibility of the cables can substantially modify EEG recordings. Furthermore, wired recordings require externalization of electrodes, which increases the risk of infection. Our study aims to address these shortcomings by developing a novel, wireless, fully enclosed and surgically stable platform for simultaneous ERG and VEP measurement in rats. There is also a scarcity of information regarding longitudinal stability of implanted electrodes [21], which we aim to address by repeated conscious recordings. Finally, we will investigate the effect of commonly used laboratory anaesthetics (ketamine:xylazine or isoflurane) on the visual recordings of rats. This methodology has potential to improve areas including longitudinal basic research, disease studies as well as preclinical drug evaluation.

## Methods

### Ethics Statement

All experiments were conducted in accordance with the Australian Code of Practice for the Care and Use of Animals for Scientific Purposes set out by the National Health and Medical Research Council. Animal ethics approval was obtained from the Animal Ethics Committee, Science Faculty, University of Melbourne. Below we detail those methodologies common to all electroretinogram (ERG) and visual evoked potential (VEP) recordings.

### General animal preparation

Long-Evans rats (male, 12 weeks-old) were housed at 21°C with a 12-hour light/ dark cycle (maximum illuminance < 50 lux). For all procedures mydriasis was achieved with one drop of both tropicamide (0.5%, Alcon Laboratories, Frenchs Forest, NSW, Australia) and topical anaesthesia with 0.5% proxymetacaine (Alcon Laboratories). Prior to ERG and VEP recordings animals were dark-adapted for 12 hours [25]. ERG/VEP experimental manipulations were performed under dim red light.

### Light stimulus

Even illumination of the retina was achieved via a Ganzfeld integrating sphere (Photometric Solutions International, Huntingdale, Victoria, Australia) as previous [26]. Signals were collected over a range of luminous energies (-5.6 to 1.52 log cd.s.m^-2^). At the dimmest intensity 80 signals were averaged, with progressively fewer averaged at brighter light levels. Interstimulus interval was progressively lengthened from 1 to 180 seconds from dimmest to the brightest light level. A twin-flash paradigm was presented at the brightest luminous energy to isolate the cone response as detailed in Nixon et al. [3]. More specifically, two flashes at 1.52 log cd.s.m^-2^ were delivered with a 500 ms inter-stimulus interval. The cone response is isolated at the second flash due to its faster recovery kinetics. At the end of ERG recording twenty VEP signals at 1.52 log cd.s.m^-2^ were collected. A single light level was used for VEP measurement to limit the duration of awake assessment to avoid stress related confounds. This protocol was utilized for all ERG/VEP collection, with the exception of experiments optimizing active and inactive electrode placements, where a single luminous energy of 0.58 log cd.s.m^-2^ was used.

### Recording equipment

Two telemetry transmitter models were utilized, single channeled CA-F40 (DataSciences International, St Paul, MN, USA) for initial electrode placement experiments and 3 channeled F50-EEE (DataSciences International) for implanted experiments. These transmitters communicate wirelessly to a receiver (RPC-1) connected to a Data Exchange Matrix (DataSciences International), which in turn is connected to a data converter (DL-10 for F50EEE, R08 for CA-F40) to interface with data collection software (ML 785, Powerlab/8SP, ADInstrument Pty. Ltd, Bella Vista, NSW, Australia). The telemetry system undergoes two stages of amplification due to the necessity to combine two different hardware systems to record ERGs. More specifically this includes the DataSciences International system, which has an amplification of x100 (F50-EEE transmitter coupled with the DL-10) and the ADInstruments Powerlab which has an amplification of x1000 (ML 785). This is in contrast to the conventional montage which only has a single stage of amplification of x1000 (ML 785 Powerlab). Thus in order to correct for the extra x100 amplification in the telemetry montage all telemetry amplitudes are divided by this factor. In this manner the amplification of both telemetry and conventional montages are equal and differences in amplitudes between the two recordings can be directly compared.

### Statistics

All group data are summarized as average ± SEM. Linear mixed model (REML) is appropriate statistical analyses for longitudinal studies as it deals with unbalanced repeated measures due to subject dropouts [27,28]. This analysis was employed to: 1. assess optimal active and inactive electrode placement 2. compare ERG parameters across time to assess implantation stability 3. compare between the anaesthetized and conscious states. Unpaired t-test was used to compare conventional chlorided silver electrodes setup to the average conscious telemetry recordings. Genstat (VSN International, Hemel Hempstead, UK) was used for statistical analysis.

### Determining optimal electrode positions

Two cohorts of dark adapted Long-Evans rats (3 months old, n = 6 each group) were utilized in experiments to determine the placement of electrodes. These experiments were conducted in rats anaesthetized with intramuscular injection of ketamine:xylazine (60: 5 mg/kg, Troy Laboratories Pty Ltd, Smithfield, NSW, Australia). At each active and inactive electrode position, ERG signals were collected at 0.58 log cd.s.m^-2^, with an inter-stimulus-interval of 3 minutes between each flash.

#### ERG active electrode placement

**Figure 1:**
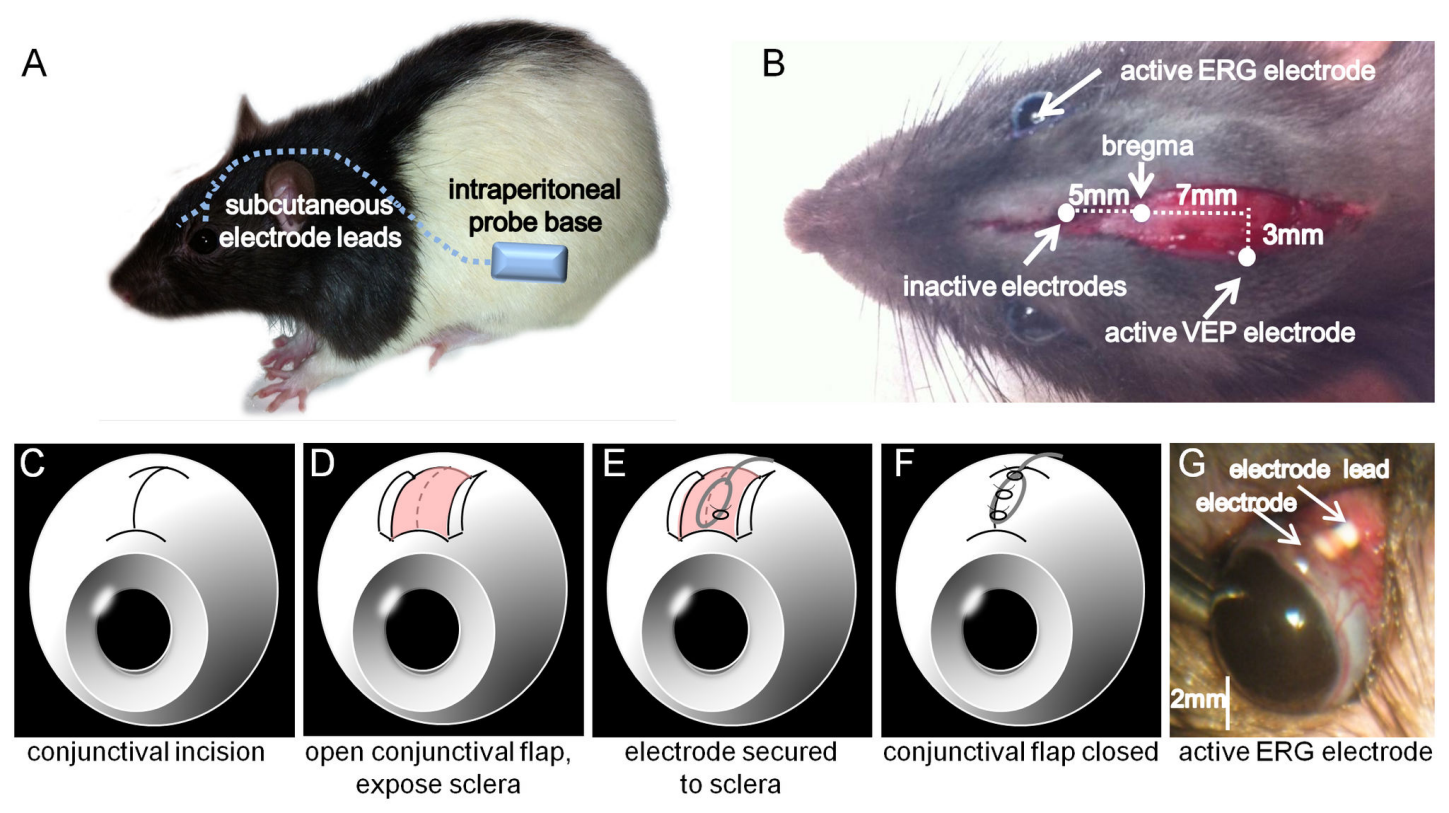
Surgical implantation of ERG telemetry probe. (**A**) Schematic illustrating location of the telemetry probe base. The electrode leads are then tunneled subcutaneously to the head. (**B**) The electrodes are attached to their respective sites; inactive ERG and VEP electrodes 5mm rostral to bregma, active VEP electrode 7 mm caudal to bregma 3mm lateral to midline, active ERG electrode to eye. (**C**-**F**) Schematics illustrating ocular microsurgical implantation of ERG active electrode. First a conjunctival flap is created (**C**) which exposes the underlying sclera (**D**). The electrode is attached to the sclera via a half scleral suture (10-10) (**E**). The conjunctival flap is then closed over the electrode (**F**). (**G**) Ocular implantation looks stable and healthy 1 week following surgical implantation. The electrode is embedded under the conjunctiva and the electrode lead can be visualized upon eyelid elevation.

As we employ a differencing system to record the ERG, both the active and inactive electrode position can influence the waveform. Using a micromanipulator (KITE-R, World Precision Instruments, Sarasota, FL, USA) to aid stabilization the inactive electrode was first placed 1 mm posterior to the limbus, on the nasal side of the sclera (Figure 1A, blue arrow). The subcutaneous inactive location on the head (Figure 1B, red arrow) was obtained by introducing a catheter (18G, Optiva IV, Smiths Medical International Ltd, Lancashire, UK) under the skin and advancing the electrode to touch the skull surface (~5 mm rostral to bregma).

**Figure 2:**
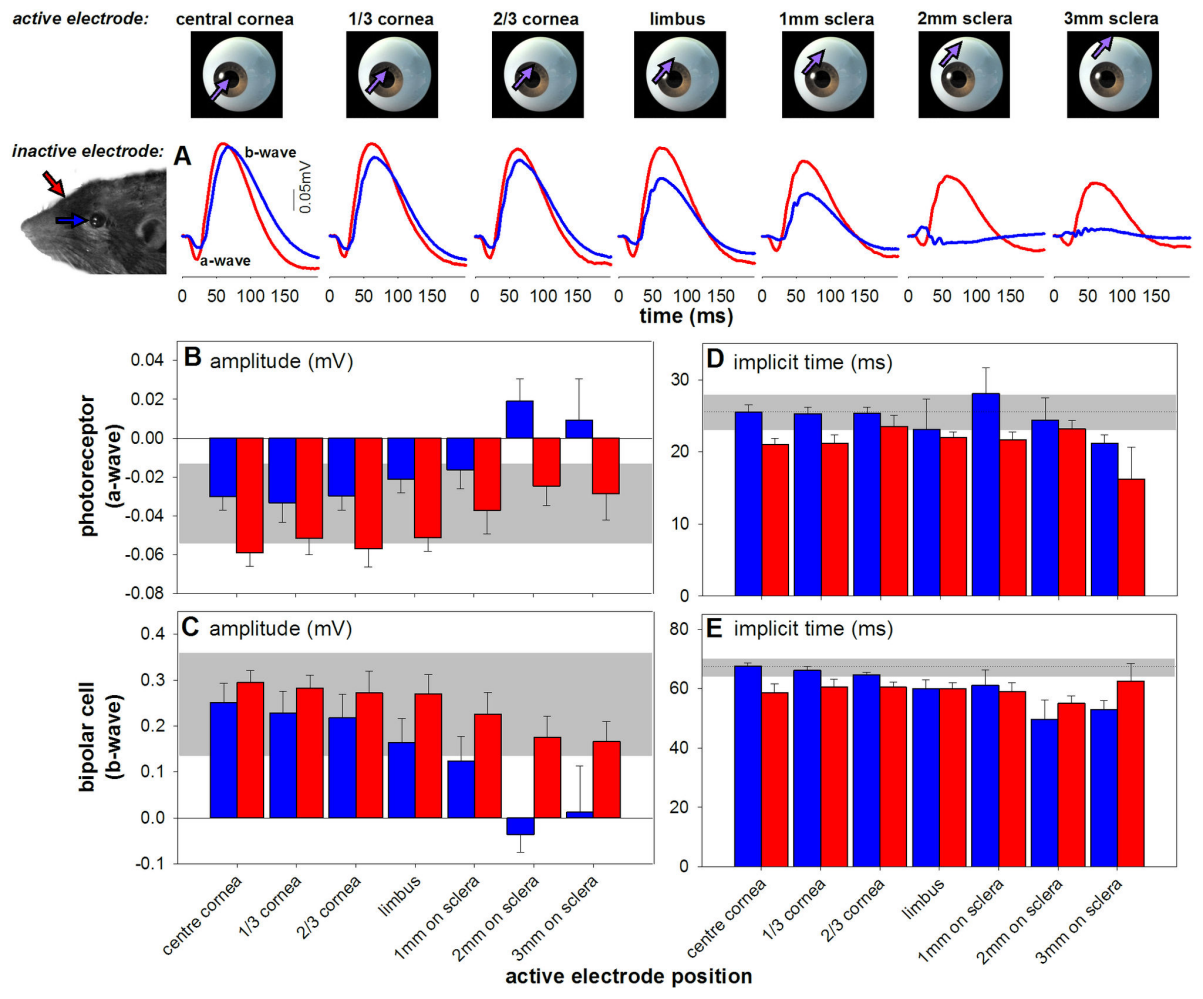
Optimizing active electrode positioning (n = 6). (**A**) Average waveforms with inactive electrode at scleral (blue) or skull (red) placement. The active electrode is placed at one of 7 locations from corneal apex to posterior sclera, along the 12 o’clock meridian. (**B**-**E**) Amplitudes (average ± SEM) and implicit time compared with conventional electrode placement (active corneal apex, inactive sclera; dashed line average; grey area 95% CI). (**B**) and (**C**) show that skull inactive electrode produced larger amplitudes than a scleral inactive location (p < 0.001). Photoreceptor and bipolar cell amplitudes were smaller (p < 0.001) with more posterior active locations. (**D**) shows that a skull inactive results in faster implicit times than a scleral inactive (p < 0.01). Implicit time did not alter with active location (p = 0.32). Placing the active electrode superiorly 1 mm behind the limbus and the inactive on the skull resulted in ERGs comparable to conventional electrode location.

At each of two inactive locations the active electrode was systematically moved from the corneal center to the posterior eye along the 12 o’clock meridian using a micromanipulator (central cornea, 1/3 cornea, 2/3 cornea, limbus, 1mm posterior limbus, 2mm posterior from limbus and 3mm posterior from limbus, see Figure 2A main text, purple arrows).

#### ERG inactive electrode placement

The active electrode placement experiment showed that signals are larger when the inactive electrode was positioned on the skull compared to the sclera. To fine-tune the optimal inactive location, a 20 mm incision was made along the rats’ cranial midline, with the underlying periosteum scrapped off to expose bregma, lambda and midline skull sutures. With the active electrode fixed at 12 o’clock, 1 mm on sclera behind limbus the inactive electrode was then systematically moved to one of four positions along the skull midline (5 mm rostral to bregma, bregma, 5 mm rostral to lambda, lambda).

### ERG analysis

ERG waveforms collected at 0.58 log cd.s.m^-2^ were analyzed for photoreceptor a-wave responses by taking the amplitude from baseline to the first trough of the first negative component. Bipolar cell response was taken as the amplitude from the a-wave trough to the largest peak (the b-wave). The implicit times taken to reach the a-wave trough and b-wave peak were also analyzed.

### Surgical implantation of telemetry transmitters

#### Animal preparation

Baseline intraocular pressure (IOP), fundus photography and ERGs under ketamine:xylazine anaesthesia were measured 1 week prior to surgery and compared to 1 week after surgery.

#### Transmitter implantation

Rats were anaesthetized using >99% isoflurane (Ceva Delvet Pty Ltd, Seven Hills, NSW, Australia) mixed with pure oxygen to give a final isoflurane concentration of 1.5-2%. The flow rate for isoflurane induction was at 3 L/min and maintained at 2 L/min throughout surgery. Respiratory rate and pinch reflex were monitored throughout surgery to ensure sufficient anaesthesia. The abdomen (animal’s right side between the leg and sternum) and the head (both approximately 30 mm x 20 mm area) were shaved then sterilized using Betadine (10% w/v povidone-iodine, Sanofi-Aventis, Australia).

A 5 mm skin incision along the midline was made on the head (between the ears) and on the abdomen. A cannula (5 mm diameter) was tunneled subcutaneously from the abdominal to head incision, enabling active and inactive wires of the F50-EEE transmitter to be passed through. The reference electrode was placed in the abdominal cavity.

The original head incision was extended to 15 mm in length, with underlying periosteum removed to reveal bregma and lambda. The rodent was placed on a stereotaxic platform (Model 900, David Kopf® Instruments, Tujunga, CA, USA), and two holes (0.6 mm diameter) were drilled (300 Series, Robert Bosch Tools, Clayton, Victoria, Australia) into the skull, without penetrating to the brain. The coordinates of the holes were 5 mm rostral to bregma on the midline (inactive electrodes) and 7 mm caudal to bregma 3 mm lateral to midline (VEP active). The electrodes were secured to the skull by lassoing the pre-made electrode loop around a stainless steel screw (diameter 0.7 mm, length 3 mm, MicroFasteners Pty. Ltd, Thomastown, Victoria, Australia) fixed into the premade holes on the skull and further secured with cyanoacrylate gel and activator (RS components, Sunshine West, Victoria, Australia).

To attach the active ERG electrodes (Figure 1C to G), a 16G guiding cannula was inserted subcutaneously from behind the eye and exteriorized in the superior conjunctival fornix. The active lead was fed from the head incision to the superior fornix. A 0.5 mm incision was made on the superior conjunctiva (12 o’clock, 1mm behind limbus) to expose the underlying sclera. The pre-looped active lead (0.2 mm diameter) was then attached to the tough outer coat of the sclera using a half-thickness scleral suture (9-0 non absorbable, Ninbo medical needles, Ningbo, China. Figure 1E). The conjunctival flaps were sutured together (9-0 non absorbable, Figure 1F) to cover the active lead and sclera. The same procedure was performed for the fellow eye. The active wire lead was then secured to the skull with cyanoacrylate gel and activator (RS components, Sunshine West, Victoria, Australia). In order to avoid tension on the eye and enable eye movements the wire at this anchoring point was not pulled taut and an extra ~1-2 mm length of wire was provided to allow for flexibility. The head wound was then closed with interrupted sutures (non-absorbable, 3-0).

The body of the transmitter was then stitched onto the intraperitoneal cavity inner wall. The initial abdomen incision was extended to ~40 mm along the midline, followed by a ~35 mm incision through the inner muscle walls and peritoneum to expose the inner cavity. The body of the transmitter was sutured (3-0, non absorbable) onto the animal’s right-side inner abdominal wall to avoid the liver. The peritoneum and muscle layer were closed via interrupted suture (non absorbable, 3-0), with the skin incision closed with a continuous suture (absorbable, 4-0).

#### Post-operative care

All animals were administered 5 mg/kg carprofen (Rimadyl, Pfizer) subcutaneously immediately following surgery and for the next 3 days. For the first 7 days post surgery, oral antibiotics (Enrofloxin, Troy Laboratories, Glendenning, NSW, Australia) were added to drinking water and Kenacomb ointment was applied (Aspen Pharma Pty Ltd, St Leonards, NSW, Australia) to the incision sites.

### Implanted ERG recordings

Animals were allowed to recover for 7 days following surgery. Conscious electrophysiology recordings were then measured at 7, 10, 14, 21 and 28 days post surgery. Animals were placed in a custom made clear plastic container similar to that used in the literature for animal restraint [29]. It has overall diameter of 60 mm with an adjustable length to snugly accommodate different sized rats. The front end of the device is tapered to minimize head movement, with perforations to allow normal breathing. There is also a perforation at the rear of the container to allow the tail to be exposed. The tapered front enables alignment of the rat’s eyes to the opening of the Ganzfeld sphere. As animals were pre-adapted to this setup (2-3 occasions prior to surgery for 30 minutes on each occasion), no obvious signs of stress were noted. This was determined by the absence of excessive muscular movements, which were continuously monitored by observation of the basal electrical trace from the eye and brain electrodes. In contrast, if the animal became uncomfortable large movement artefacts would be immediately apparent.

On days 7 and 14 post surgery, following conscious recordings in the morning, ERG and VEP protocols were recorded under anaesthesia using either intramuscular injection of ketamine:xylazine (60: 5 mg/kg) or inhalant isoflurane (2% at flow rate 1.5-2 L/min) in the afternoon. The anaesthetic used on day 7 was chosen randomly with the other one applied on day 14.

### Conventional ERG and VEP

As previously reported, ERGs [26] were recorded by placing a blunt, chlorided silver electrode on the corneal apex (active) and another tapered chlorided silver electrode tucked into the temporal fornix (inactive) on the anaesthetized rat. VEPs [30] were measured by implanting a stainless screw over rat’s visual apex, which connects to the active electrode. The inactive (chlorided silver) electrode is hooked around rat’s incisor. A stainless needle inserted into the tail acts as the ground electrode in both setups.

### ERG Analysis

#### Photoreceptoral response

The leading edge of the scotopic a-wave can be modeled with a delayed Gaussian based on the model of Lamb and Pugh, formulated by Hood and Birch [31,32], 

$$P3(i,t)=Rm_{P3}\cdot [1-\exp\left(-i\cdot S\cdot {\left(t-t_{d}\right)}^{2}\right)], t > td$$ (1)

where the photoreceptoral response (mV) for a given luminous energy (*i*, log (cd.s.m^-2^)) can be described as a function of time (*t*, s), saturated photoreceptoral amplitude (Rm_P3_, mV), photoreceptoral sensitivity (*S*, log(m^2^.cd^-1^.s^-3^)) and delay (*t*_*d*_, s) ,which is mainly due to lag in the recording equipment [33,34]. The *t*_*d*_ values were fixed to their group average determined by conducting bootstrap analysis. This returned an average *t*_*d*_ of 7.4ms for telemetry data and 4.75 ms for silver/silver chlorided ERG. Photoreceptoral amplitude (*Rm*_*P3*_) and sensitivity (*S*) were then optimized by minimizing sum-of-square merit function using the Solver module in Excel (Microsoft™, Redmond, WA, USA) across an ensemble of the top two luminous energies (1.20, 1.52 log cd.s.m^-2^).

#### Bipolar cell response

The modeled photoreceptoral response (P3) was subtracted from the raw ERG to give the putative P2 (bipolar cell) response. Whilst the P3 model does not account for the deactivation of phototransduction, its subtraction to isolate a putative P2 provides a more accurate description of the bipolar cell response than conventional peak-to-peak amplitudes [35]. The function was then modeled with a saturating hyperbolic function given by Equation 2,

$$V(i)=V_{\max}\frac{i}{i^{}+k^{}}$$ (2)

where the P2 amplitude (*V*, mV) is expressed as a function of luminous energy (*i*, log cd.s.m^-2^), bipolar cell amplitude (*V*_max,_ mV) and semi-saturation constant (*k*, log cd.s.m^-2^). For consistency with photoreceptoral analysis, the semi-saturation constant (K, cd^-1^.s^-1^.m^2^) is expressed as inverse of the antilog to give the bipolar cell sensitivity parameter. V_max_ and k were minimized using sum-of-square merit function using the Solver module in Excel.

#### Cone analysis

The cone response is assayed for the a-wave amplitude (a-wave_min_), and implicit time (a-wave_it_). The rat cone a-wave receives major contributions from the cone photoreceptors as blocking post-receptoral contributions shows minimal influence on this waveform (see Figure 14C from Bui et al [36]). The cone b-wave is also assayed for amplitude (b-wave_max_) and implicit time (b-wave_it_) which has been shown to reflect bipolar cell responses.

#### VEP analysis

P1, N1 and P2 amplitudes and implicit times are extracted for each VEP waveform. For consistency, these three landmarks are defined at the first peak, which is most relevant for P2 analysis as several peaks manifest following the N1. This is in accordance with VEP analysis conducted in the literature [37,38].

## Results

It is important to establish that the surgical procedure has no adverse effect on the animal. Visualization of the retina showed no visible changes in gross retinal integrity and blood supply to the eye post surgery. Comparison of intraocular pressure (IOP) before and 7 days after surgery returned no significant difference (before 13 ± 1 mmHg, after 13 ± 2 mmHg, p = 0.68). Likewise, ERG parameters measured via conventional, anaesthetized, chlorided silver electrode montage (the current gold standard) before and after surgery (photoreceptor amplitude before surgery -0.332 ± 0.055 vs. after -0.356 ± 0.030 mV, p = 0.74; photoreceptor sensitivity 1270 ± 401 vs. 1024 ± 88m^2^.cd^-1^.s^-3^, p = 0.61; bipolar cell amplitude 1.246 ± 0.192 vs. 1.217 ± 0.093 mV, p = 0.93; bipolar cell sensitivity 85 ± 19 vs. 105 ± 19 cd^-1^.s^-1^.m^2^, p 0.49) indicate no adverse effects from our surgical manipulation. These data indicate that the surgical implant procedure causes minimal ocular injury.

### Optimal active electrode placement

Figure 2A shows ERG waveforms vary with active and inactive electrode position (sclera, blue vs. skull, red). It appears that signal sizes are consistently larger when the inactive electrode is placed on the skull compared to the sclera, as confirmed in Figure 2B (photoreceptor) and 2C (bipolar cell) (p < 0.001). As active electrode moves more posterior from the corneal apex there is significant amplitude reduction (p < 0.001). It is instructive to compare changes in signal size obtained from the different montages shown in Figure 2A with conventional electrode placement (inactive at sclera, active at central cornea). This demonstrates that for all active electrode positions with the inactive on the skull, there was no significant attenuation of signal size compared with the conventional placement using the telemetry montage (grey box, 95% confidence limits).

We observed significantly slower photoreceptor implicit times with the inactive on the sclera compared to on the skull (Figure 2D; p < 0.01). However, there was no difference across different active electrode locations (p = 0.19). Bipolar cell implicit time showed little effect of electrode placement except it was significantly faster with the active electrode at 2 mm on sclera compared to all other active positions (all p < 0.05).

### Optimal inactive electrode placement

**Figure 3:**
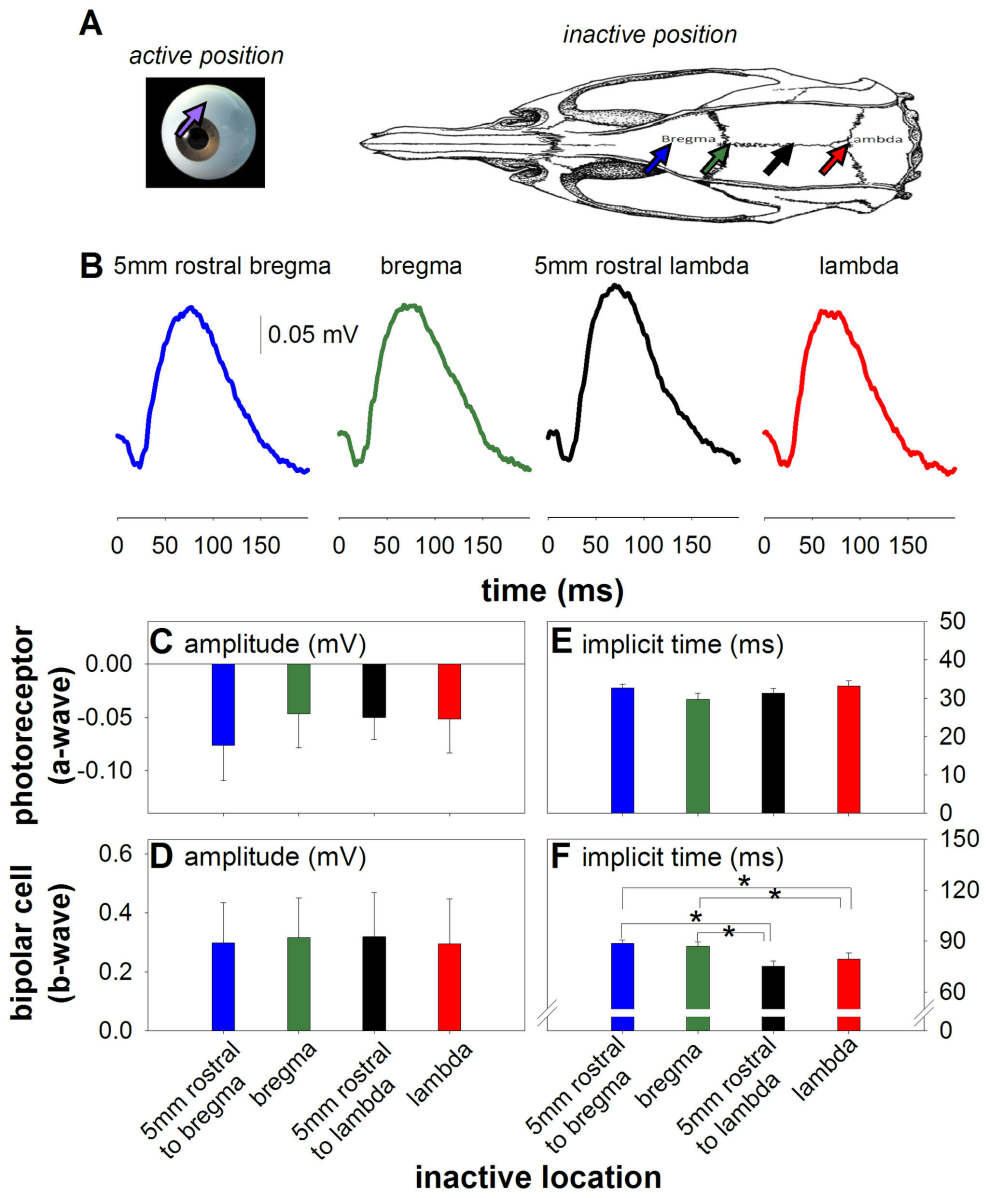
Optimizing inactive skull electrode positioning (n = 6) (A) Schematic of inactive electrode position (B) Average waveforms where the active electrode is optimally placed for telemetry (superior 1 mm behind limbus) and the inactive electrode is placed at 4 positions on the head. Photoreceptor amplitude (**C**) bipolar cell amplitude (**D**) and photoreceptor timing (**E**) are not altered with inactive location. Bipolar cell timing (**F**) is slower when the inactive electrode is at bregma and 5mm rostral to bregma. * indicates statistical difference (p < 0.05).

Figure 2 shows that the largest signal amplitudes are found with the inactive placed on the head. However, it is possible that a head location can become contaminated by cortical activities. To investigate optimal inactive electrode location, the active electrode was fixed (1 mm posterior to the limbus) and the inactive was systematically moved across four locations on the skull (Figure 3A). Figure 3B shows that there was no significant difference in photoreceptor (Figure 3C, p = 0.15) and bipolar cell amplitudes (Figure 3D, p = 0.67) across the four positions. This was also the case for photoreceptor implicit time (Figure 3E, p = 0.33). Bipolar cell implicit times are significantly different (p < 0.01), with post hoc tests showing those implicit times with the inactive at 5 mm rostral to bregma and bregma are slower compared to 5 mm rostral to lambda and lambda.

### Stability of conscious ERG signals

**Figure 4:**
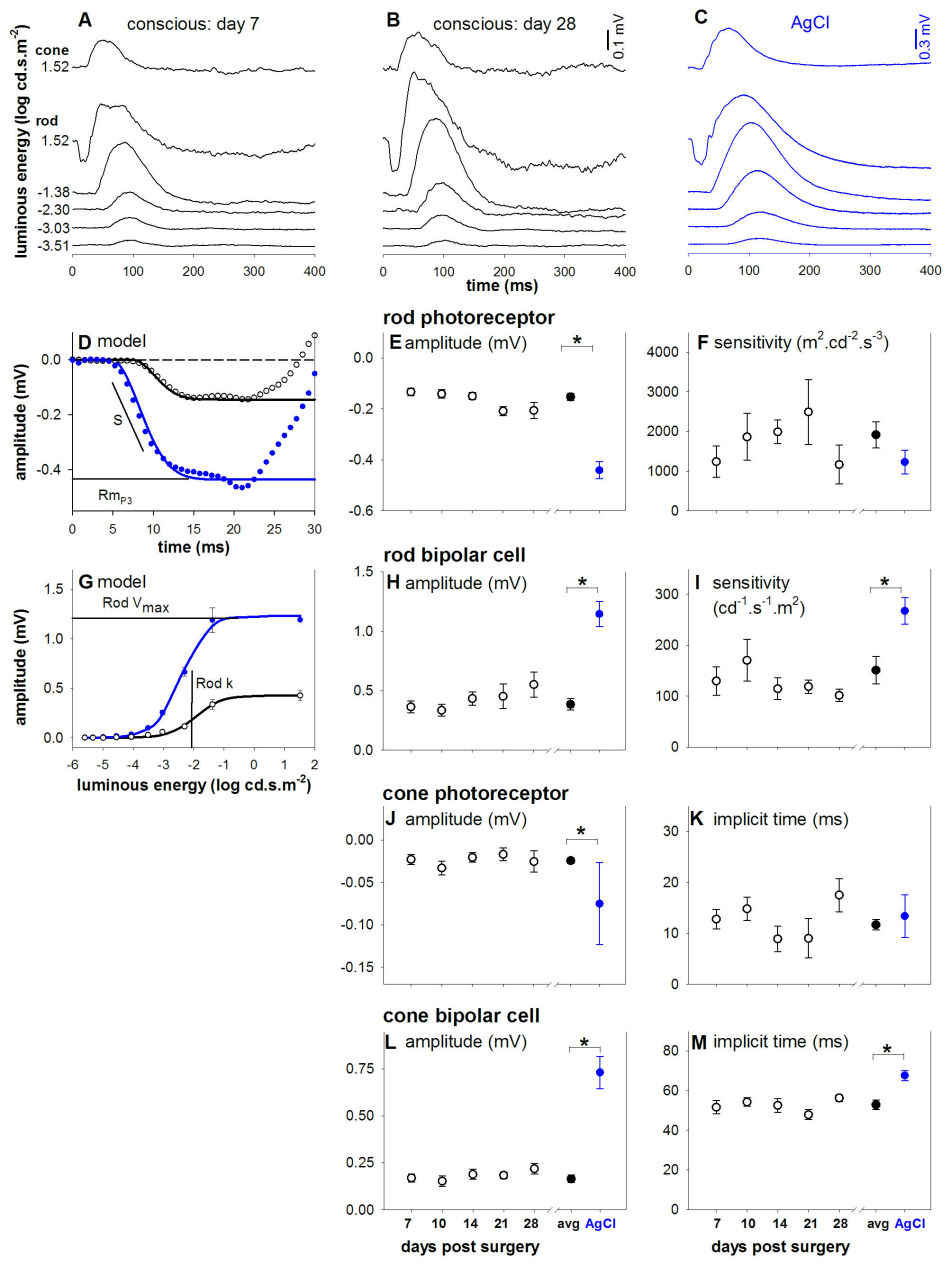
Electroretinography using telemetry electrodes results in stable repeatable measurements. (**A**-**C**) Group average ERG waveforms across a wide range of luminous energies. Conscious electroretinography (black, day 14 (A) and 28 (B) post surgery) is compared against the gold standard ERG montage (blue, AgCl electrodes under ketamine:xylazine anaesthesia (**C**)). Note the different scale bars. (**D**) The rod photoreceptor response was modeled (black: awake telemetry, blue: conventional AgCl setups) with a delayed Gaussian over an ensemble of two luminous energies (1.20 and 1.52 log cd.s.m^-2^, only 1.52 log cd.s.m^-2^ shown), which is then derived into amplitude (**E**) and sensitivity (**F**) parameters. (**G**) The rod-isolated bipolar cell energy response was modeled with a hyperbolic function (black: awake telemetry, blue: conventional AgCl setups), from which amplitude (**H**) and sensitivity (**I**) parameters are extracted. Panels (**J**, **K**) show cone photoreceptor parameters with (**L**) and (**M**) showing cone bipolar cell indices. Panels (**D**-**M**) illustrate the stability of conscious ERG recordings from 7 to 28 days after surgical implantation (unfilled black, left scale bar). All rod and cone amplitude and timing parameters showed no significant change across time after surgical implantation. The average conscious ERG recordings (filled black, day 7 to 28 post-surgery, left scale bar) is then compared to the gold standard electrode montage (blue). * indicates statistical difference (p < 0.05).

Figure 4 assesses the longitudinal stability of telemetric ERG implantations and compares conscious ERG parameters to conventional, anaesthetized recordings with chlorided silver electrodes. Figure 4A and B show that conscious telemetry exhibits similar ERG waveform characteristics to the gold standard anaesthetized montage (Figure 4C. chlorided silver electrodes, ketamine:xylazine anaesthesia). Not surprisingly, the anaesthetized set-up returns quieter waveforms than does telemetry (see Figure S1 in supplementary material for more detail regarding signal-to-noise characteristics and Figure S2 for coefficient of variation). Nevertheless, telemetry shows a similar intensity response relationship. At dim intensities the ERG contains a positive bipolar cell derived b-wave, which grows in amplitude with flash energy. The brightest intensities show the classic ERG configuration: a negative a-wave followed by a positive b-wave.

Figure 4D shows that the photoreceptor response (unfilled circles) returned from telemetry (black) and gold-standard montage (blue) can both be described using the well accepted model of rod phototransduction (solid lines; Equation 1). The most obvious difference in anaesthetized waveforms is the faster onset of the response (2.6 ms earlier than conscious telemetry recordings), which is likely due to differences in recording hardware, as studies have shown that the delay in timing largely reflects lag in the equipment [33,34]. The average photoreceptor amplitude (Figure 4E) and sensitivity (Figure 4F) are stable over the period of 1 month as assayed by recordings on 7, 10, 14, 21 and 28 days post surgery. Figure 4G shows that both telemetry and chlorided silver electrode bipolar cell intensity-response functions are well described using a hyperbolic relationship. Rod bipolar cell amplitudes and (Figure 4H) and sensitivities (Figure 4I) are stable over 28 days. The stability of conscious ERG parameters was confirmed for all parameters across time (linear mix model (REML) time effect: p = 0.14 to 0.67), and is confirmed by a stable signal-to-noise ratio (Figure S1 in supplementary material).

As there is no effect of time after surgery with conscious telemetry, recording parameters were averaged across measurement times and compared against the gold standard chlorided silver anaesthetized montage (blue circles). The amplitudes of all parameters are significantly larger in the conventional AgCl setup compared with telemetry (Figures 4E, 4H, 4J and 4L; p < 0.05: NB different scales on left and right axes). In terms of sensitivity, there was no difference in photoreceptoral sensitivity (Figure 4F rod S, p = 0.18), but a significantly less sensitive bipolar cell response (Figure 4I, rod K p < 0.05) in telemetry compared with AgCl electrode recordings. Cone photoreceptor implicit time was not different (Figure 4K, cone a-wave_it_, p = 0.63), whereas cone bipolar cells show faster implicit times (Figure 4M, cone b-wave_it_, p < 0.05) with the telemetry montage in contrast to the delayed rod response.

### Conscious versus anaesthetized ERGs

**Figure 5:**
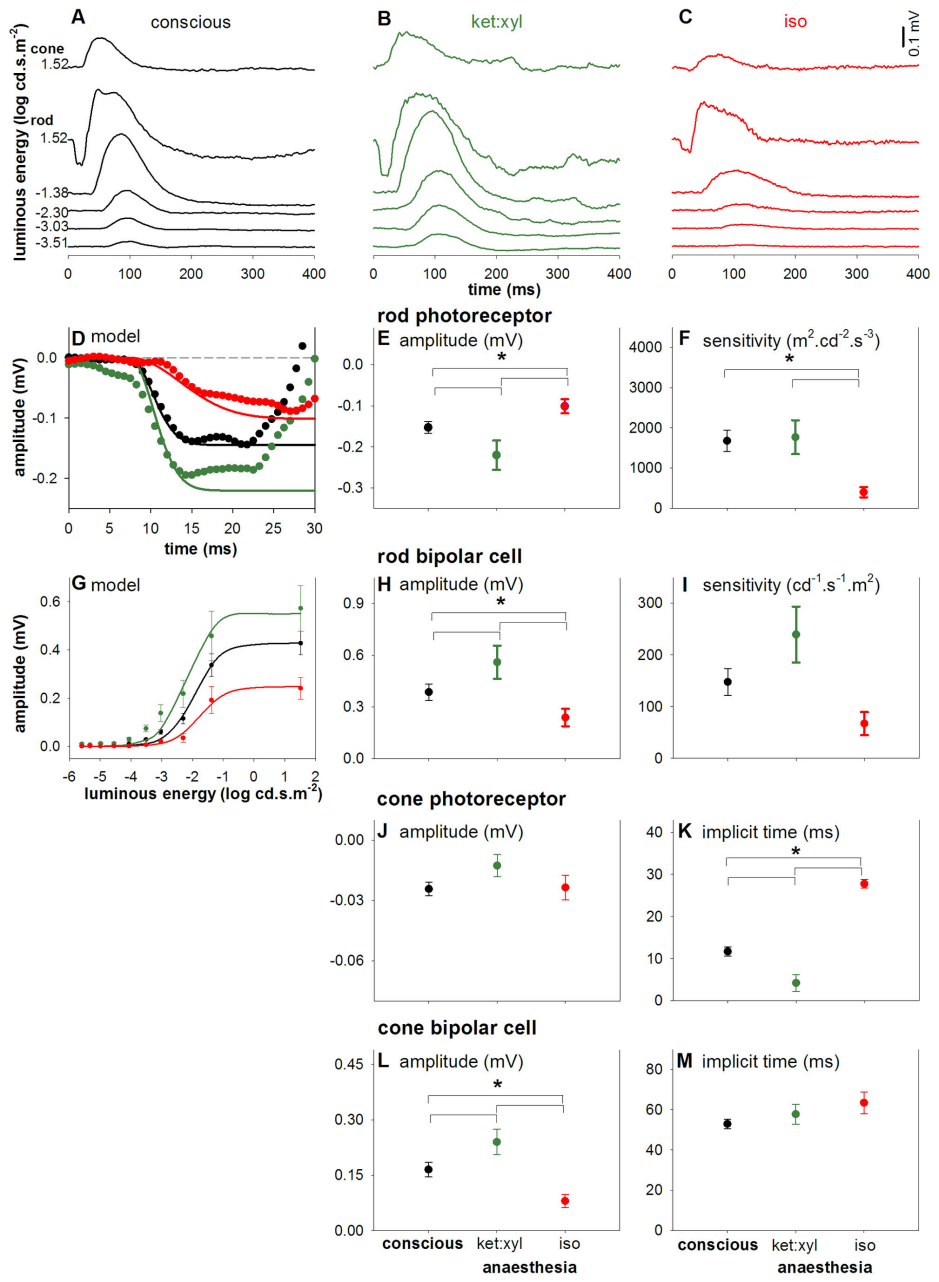
Effect of anaesthesia on the electroretinogram (A) Group average ERG waveforms across a wide range of luminous energies. Conscious ERGs (black, average day 7 to 28 post-surgery) are compared against ERGs conducted under ketamine:xylazine (**B**, green) and isoflurane (**C**, red) anaesthesia. (**D**) The rod photoreceptor response was modeled with a delayed Gaussian in which amplitude (**E**) and sensitivity (**F**) parameters are derived. (**G**) The rod-isolated bipolar cell energy response was modeled with a hyperbolic function, from which amplitude (**H**) and sensitivity (**I**) parameters are extracted. Panels (**D**-**M**) illustrate the effect of anaesthetic agents on rod and cone parameters of the ERG. Ketamine:xylazine anaesthesia increased amplitudes of rod photoreceptors (**E**) and rod and cone bipolar cells (**H**, **L**) and a faster cone photoreceptoral timing (**K**). In contrast, isoflurane causes a reduction in all ERG amplitudes except cone photoreceptor (**J**). Isoflurane also resulted in slowed kinetics of all photoreceptor parameters (**F**, **K**). * indicates statistical difference (p < 0.05).

Figure 5 compares the effect of two types of commonly used laboratory anaesthetics on the conscious ERG. Group average waveforms (Figure 5A-C) show that ketamine:xylazine (green, Figure 5B) increases but isoflurane (red, Figure 5C) dampens ERG responses. All conscious parameters are averaged across time as previous analysis showed no significant time effect (Figure 4). Post-hoc analysis reveals that isoflurane decreases rod photoreceptor, rod and cone bipolar cell amplitudes, reduced rod photoreceptor sensitivity (see Figure S3 for rod implicit time data) and delayed cone photoreceptor implicit time. On the other hand, ketamine:xylazine significantly increased rod photoreceptor, rod bipolar cell and cone bipolar cell amplitudes but decreased cone photoreceptor implicit time compared to conscious state (all p < 0.05).

### Stability of conscious VEP signal and effect of anaesthesia

**Figure 6:**
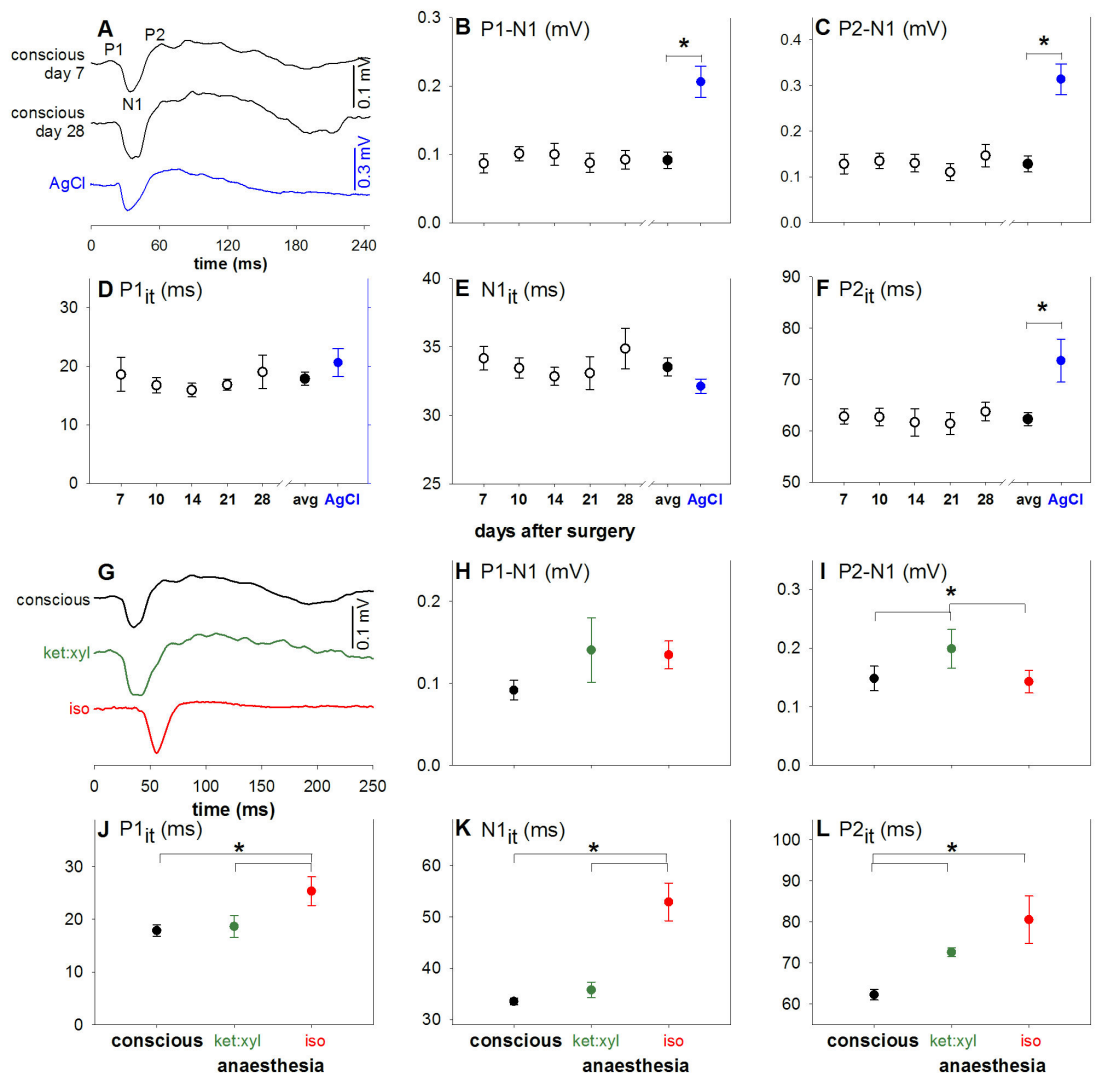
Stability of conscious visual evoked potentials and the effect of anaesthesia. (**A**-**F**) illustrate the stability of conscious VEP measurements across 7 to 28 days post-surgery (unfilled black: awake day recordings, filled black: average awake recordings across day7 to 28 post-surgery, blue: gold standard montage), note different scale bars. (**A**) Group average VEP waveforms (black, day 7 and 28 post surgery) are compared against the gold standard ERG montage (blue, stainless steel electrodes under ketamine:xylazine anaesthesia). Panels (**B**-**F**) illustrate the stability of awake ERG recordings from 7 to 28 days after surgical implantation (black, left scale bar). There is no significant change in VEP parameters across time. Thus the average awake VEP recordings (filled black, day 7 to 28 post-surgery, left scale bar) are then compared to the gold standard electrode montage (blue, right scale bar). Amplitude parameters of the VEP are larger in gold standard montage compared to awake telemetry recordings (**B**, **C**) and the P2it (**F**) is faster. (**G**-**L**) Illustrate the effect of anaesthesia on the VEP (black: awake, green: ketamine:xylazine, red: isoflurane). Ketamine:xylazine causes increased P2-N1 amplitudes when compared with conscious recordings (**I**). Isoflurane causes decreased P2-N1 amplitude (**I**) and slowed timing (**J**-**L**). * indicates statistical difference (p < 0.05).

Figure 6A to F examines the stability of telemetry implanted VEPs in conscious animals as well as the effect of anaesthesia. No significant effect for time after surgery was found for any VEP parameter across the 7 to 28 day period of recording (Figure 6B to F unfilled circles, p = 0.2 to 0.93), which reflects stability of the surgical implantation. Unpaired t-test between averaged conscious VEP (filled black circle) and the conventional anaesthetized setup (filled blue circle) revealed significantly larger amplitudes in both P1-N1 and P2-N1 (p < 0.05) for the conventional AgCl setup. In terms of timing, P1 and N1 implicit times are similar (p = 0.33 and 0.36 respectively), but P2_it_ was slower (p < 0.05) in conscious compared with conventional anaesthetized recordings.

Figure 6G to L compares the effect of different anaesthesia regimen on the VEP and shows that conscious (black) recordings are not dissimilar to ketamine:xylazine (green). However, isoflurane (red) produces slower waveforms and no obvious oscillations after P2. Analysis of VEP parameters (Figure 6H to L) shows that the P1-N1 amplitude was not different (p = 0.22), whereas the P2-N1 (p < 0.05) was significantly larger with ketamine:xylazine. All timing parameters showed a marked delay with isoflurane compared to conscious recordings (Figure 6J to L, all p < 0.01) whereas ketamine:xylazine only slowed P2 implicit time (Figure 6L, p < 0.05).

## Discussion

This is the first study to successfully implant wireless technology to assess conscious retinal and cortical electrophysiology simultaneously in rats. Our ERG and VEP implants are stable for at least 28 days and both signals can be recorded as early as 7 days after surgery.

### Electrode placement

The decision to implant active ERG electrodes 1mm posterior to the limbus derives from a compromise between animal comfort and signal strength, whereas the location of active epidural screw VEP electrodes has been shown to produce the largest and most stable VEPs [30]. For the ERG active electrode, as the cornea contains sensory nerves [39], to implant at this location would cause discomfort and also increase the risk of corneal infection and electrode detachment. We therefore chose to implant ERG active electrodes 1 mm from the limbus on the sclera. This active position when coupled with an inactive skull location gave ERG responses similar to those with the active electrode placed on the central cornea (Figure 2). Valjakka [22] found larger rat ERG signals when the active electrode was on the cornea instead of sclera. Their smaller signals were recorded with the active placed posterior to the equator of the rat eye, consistent with our findings (Figure 2).

Various techniques have been adopted to implant chronic ERG active electrodes in the rat such as suturing wires under the eyelid [21]. This approach produced small and variable amplitudes. These confounds may be due to a more posterior electrode location and eyelid blinking respectively. Alternative approaches include inserting a stainless steel screw laterally through the skull until it touches the posterior globe [40] or inserting a silver ball attached to copper wire to touch the posterior sclera [22]. With these approaches the final electrode location is difficult to visualize and define precisely. Our technique of securing the electrode to the easily visualized anterior sclera makes it easy to standardize location between animals. We believe this produces more stable signals as will be discussed below.

For inactive electrode location, we chose to anchor it on the skull, 5 mm rostral to bregma, a location commonly used in electroencephalography recordings [41] as there should be no brain structure directly underneath, giving minimal contamination by other brain potentials. We infer this may contribute to differences in b-wave implicit times in Figure 3F as intrusion of the VEP waveform on the ERG inactive electrode would result in the faster implicit times seen in lambda placements. That there is not a large amplitude difference at these lambda locations may be due to the magnitude difference between ERG and VEP. More specifically, VEP recordings are approximately one-fifth of ERG amplitudes. Hence any VEP contribution to ERG across different skull positions may not be large enough to manifest in the ERG amplitudes.

### Comparison to current gold standard

As this is a novel adaptation of wireless technology, a comparison was undertaken against the current rodent gold-standard montage (a corneal active and scleral ring inactive montage with chlorided silver electrodes). Figure 4A-C shows that the telemetry waveforms (Figure 4A-B) resemble those returned from chlorided silver electrodes (Figure 4C) across a range of light intensities. It is worth noting that telemetry waveforms do not exhibit oscillatory potentials due to the bandwidth (1-100Hz) of the DSI telemetry probe (F50-EEE). This could be overcome by using a different telemetry probe (i.e. CA-F40 bandwidth 1-200Hz) however such an approach sacrifices the number of channels available for recording (one instead of three). As our study aimed to simultaneously monitor ERG and VEP we deemed the F50-EEE to be more appropriate.

ERG and VEP amplitudes and kinetics recorded with conventional AgCl electrodes are different when compared to those under the telemetry montage. These discrepancies can be attributed to various factors, such as different electrode materials [42] and recording hardware as well as the use of anaesthesia (Figures 5 and 6). The amplitude difference between the two montages is consistently 3-fold for all ERG and VEP parameters. This magnitude difference is not uncommon between different electrode materials and placements [43]. Despite the parameter differences (albeit scalable), we believe our novel telemetry methodology represents responses least contaminated by anaesthesia. It is also noteworthy that the signal to noise ratio achieved using the telemetry montage for conscious recordings is 1/3 of anaesthetized recordings using conventional AgCl (see Figure S1). To offset this disadvantage more signal averaging can be employed, as experiments are not limited by the duration of safe and effective anaesthesia.

### Repeatability of conscious ERG/VEP recordings

As no studies have considered wireless ERG and VEPs we compare our repeatability to wired recordings. Only one paper has investigated the repeatability of ERG/VEP recordings. Szabo-Salfay et al [21] found that wired ERG and VEP signals grow between day 3 and 1 month after surgery. Our study found good stability from day 7 to day 28 post surgery. The discrepancy in stability between our and the previous study may reflect different surgical implantation techniques or the possibility that the animals may still be recovering from surgery at the first recording session (3 days post surgery) in the Szabo-Salfay study. This surgical recovery can explain the subsequent growth in amplitude reported by the authors. Whilst scarring at the surgical site may change impedance over time, the stability of the signals in our recordings suggests that this effect is negligible.

### Anaesthesia effect

It is expected that ERG and VEP responses would be affected by anaesthesia, as the receptors targeted in their analgesic and sedative efficacy are also located in the eye and cortical visual pathways. Guarino et al [11] found urethane altered ERG and VEP responses. We extended this investigation to show that two commonly used laboratory anaesthetics (ketamine:xylazine mixture or isoflurane) altered ERG and VEP responses.

Compared to the conscious state, isoflurane anaesthesia (Figures 5 and 6) produced overall attenuation of the ERG, with marked timing delays in both ERG and VEP. In humans, Tremblay and colleagues [12] found a decrease in rod b-wave amplitudes and slowing of all ERG parameter timings with isoflurane administration, mirroring our findings in rodents. Isoflurane has been shown to act on NMDA, GABA and glycine receptors. This wide-ranging effect may underlie our findings of extensive dampening of ERG features. Furthermore, studies have shown that intravitreal injection of GABA and glycine into rodent eyes [36,44] resulted in smaller and slowed ERG waveforms. It was surprising that 2% isoflurane did not dampen the VEP amplitude as much as was reported by Jehle and colleagues [10]. Whilst the reason for this discrepancy is not clear, isoflurane may have different effects on the generator of the flash VEP - used in this study - compared with the steady state response elicited in by Jehle et al [10]

Given that neither ketamine nor xylazine show efficacy on these NMDA or GABA receptors, this may explain the more subtle ERG and VEP effects with ketamine and xylazine. Jehle and colleagues [10] have shown that a light dose of ketamine:xylazine (65: 7 mg/kg, similar to this study 60: 5 mg/kg) produced increased VEP amplitudes, consistent with our findings. These data suggest that a light dose of ketamine:xylazine better represents conscious visual electrophysiology than isoflurane.

## Conclusions

Our novel wireless approach offers a robust platform that can be applied to a broad range of research. Our ERG and VEP recording techniques can be implemented across a wide range of animal models, with the caveat being that implanting on a species with smaller eyes and thus a thinner sclera would be more technically challenging. The capacity to measure simultaneous, *in vivo*, conscious brain and eye electrophysiology affords real-time, uncontaminated assessments of the visual pathway. Studies requiring long or repeated periods of anaesthesia are associated with increased risk of anaesthetic complications. This has hampered longitudinal assessment in many rodent models of disease, such as diabetes, glaucoma as well as other neurodegenerative conditions. Given that previous preclinical work on rats have been performed under anaesthesia, which we have shown confounds ERG and VEP responses, we believe our research platform will improve translation from the bench to the bed-side as it better replicates clinical recordings. Applicability of the eye as a functional biomarker has been limited due to contamination associated with anaesthetic drug interactions and/or stress. This platform will make prolonged studies of drug pharmacodynamic responses lasting hours to weeks and even months feasible.

## Supporting Information

Figure S1 **The telemetry system demonstrates stable SNR over time (unfilled circles, average SNR ±SEM).** Conventional AgCl SNR (filled blue) is significantly larger than telemetry average SNR (filled blue).
(DOCX)

Figure S2 **Coefficient of variation (CoV) of ERG amplitude parameters across days of awake testing (unfilled bars), average (± SEM) CoV of the 5 recording sessions (filled black), compared to CoV under anaesthesized conditions with conventional AgAgCl electrodes (filled blue).**
(DOCX)

Figure S3 **A plots average (±SEM) rod photoreceptor implicit times under conscious (black filled), ketamine:xylazine (green) and isoflurane (red) states measured with the telemetry montage for rod photoreceptor implicit time at 1.52 log cd.s.m^-2^.** Implicit times are also plotted for rod bipolar cell at 1.52 (**B**) and -1.38 (**C**) log cd.s.m^-2^ * indicates statistical difference (p<0.05).
(DOCX)
