# Supplementary material for: Conscious Wireless Electroretinogram and Visual Evoked Potentials in Rats
Source: PLoS One. 2013 Sep 12;8(9):e74172. doi: 10.1371/journal.pone.0074172 (PMC3771909; doi:10.1371/journal.pone.0074172)
Supplement: Figure S1 — The telemetry system demonstrates stable SNR over time (unfilled circles, average SNR ±SEM). Conventional AgCl SNR (filled blue) is significantly larger than telemetry average SNR (filled blue). (DOCX) [file pone.0074172.s001.docx]

**Figure S1.** The telemetry system demonstrates stable SNR over time (unfilled circles, average SNR ±SEM). Conventional AgCl SNR (filled blue) is significantly larger than telemetry average SNR (filled blue).

Figure S1 shows signal to noise (SNR) ratio of awake telemetry ERG signals across the days of measurement (unfilled black) and compares the group average (filled black) to that obtained from conventional, anaesthetised AgAgCl recordings (filled blue). Signal size was taken as the largest amplitude (PII_amp_) and noise was taken as the peak-to-peak amplitude over a 10 ms window of basal recording without light stimulation, and thus can be interpreted as “movement artefact noise”. REML analysis revealed no significant time effect from 7 to 28 days post surgery, indicating a stable SNR (p = 0.49). Unpaired t-test between SNR averaged across the 5 awake recording sessions and conventional AgCl showed greater SNR in conventional recordings (p < 0.05). This could be attributed to a larger signal size associated with silver chloride electrode materials and/or decreased noise levels with the introduction of anaesthesia.
