# Supplementary material for: Conscious Wireless Electroretinogram and Visual Evoked Potentials in Rats
Source: PLoS One. 2013 Sep 12;8(9):e74172. doi: 10.1371/journal.pone.0074172 (PMC3771909; doi:10.1371/journal.pone.0074172)
Supplement: Figure S2 — Coefficient of variation (CoV) of ERG amplitude parameters across days of awake testing (unfilled bars), average (± SEM) CoV of the 5 recording sessions (filled black), compared to CoV under anaesthesized conditions with conventional AgAgCl electrodes (filled blue). (DOCX) [file pone.0074172.s002.docx]

**Figure S2.** Coefficient of variation (CoV) of ERG amplitude parameters across days of awake testing (unfilled bars), average (± SEM) CoV of the 5 recording sessions (filled black), compared to CoV under anaesthesized conditions with conventional AgAgCl electrodes (filled blue).

Figure S2 shows coefficient of variation (CoV) on different days of awake testing and compares this against the conventional montage under anaesthesia. Conventional recordings show lower CoV than awake recordings, most likely due to decreased movement variability with anaesthesia (corresponding to greater SNR). Nevertheless, the awake telemetry montage shows CoV across the various measurement days that are comparable to the AgAgCl montage. One exception to this is for cone photoreceptor amplitude, where CoV in the AgAgCl case is lower. This variability may arise from the small signal sizes associated with the cone a-wave.
