# Supplementary material for: Conscious Wireless Electroretinogram and Visual Evoked Potentials in Rats
Source: PLoS One. 2013 Sep 12;8(9):e74172. doi: 10.1371/journal.pone.0074172 (PMC3771909; doi:10.1371/journal.pone.0074172)
Supplement: Figure S3 — A plots average (±SEM) rod photoreceptor implicit times under conscious (black filled), ketamine:xylazine (green) and isoflurane (red) states measured with the telemetry montage for rod photoreceptor implicit time at 1.52 log cd.s.m-2. Implicit times are also plotted for rod bipolar cell at 1.52 (B) and -1.38 (C) log cd.s.m-2 * indicates statistical difference (p<0.05). (DOCX) [file pone.0074172.s003.docx]

**Figure S3.** **A** plots average (±SEM) rod photoreceptor implicit times under conscious (black filled), ketamine:xylazine (green) and isoflurane (red) states measured with the telemetry montage for rod photoreceptor implicit time at 1.52 log cd.s.m^-2^. Implicit times are also plotted for rod bipolar cell at 1.52 (**B**) and -1.38 (**C**) log cd.s.m^-2^ * indicates statistical difference (p<0.05).

Figure S3 plots rod photoreceptor and bipolar cell implicit times over for average conscious, under ketamine:xylazine or isoflurane with the telemetry system. REML returned significantly slowed rod photoreceptor implicit time under isoflurane compared to the two other states (p<0.05). However, as Figures S3B and C show, rod bipolar cell implicit time results is dependent on the luminous energy. Therefore the sensitivity parameter is a better indicator of the overall dynamics of photoreceptoral and bipolar cell responses across the range of luminous energies.
